# Analysis of complete mitochondrial genome of *Etisus anaglyptus* (Arthropoda, Decapoda, Xanthidae) with phylogenetic consideration

**DOI:** 10.1080/23802359.2018.1443038

**Published:** 2018-02-24

**Authors:** Mustafa Zafer Karagozlu, Thinh Do Dinh, Van Quan Nguyen, Chang-Bae Kim

**Affiliations:** a Department of Biotechnology, Sangmyung University, Seoul, Korea;; b Institute of Marine Environment and Resources, Vietnam Academy of Science and Technology, Haiphong, Vietnam

**Keywords:** Arthropoda, Decapoda, Xanthidae, complete mitochondrial genome, *Etisus anaglyptus*

## Abstract

The complete mitochondrial genome is sequenced and analyzed from a xanthid crab *Etisus anaglyptus,* which is the first complete mitochondrial genome for the genus. The mitochondrial genome length of *E. anaglyptus* is 16,435 bp and it is composed of 13 protein coding genes, two ribosomal RNA genes, and 22 tRNA genes. The structure and gene orientation of the mitochondrial genome is identical with the other brachyuran records. Furthermore, phylogenetic relationships of the infraorder Brachyura evaluated by mitochondrial protein coding genes. The phylogenetic study showed that *E. anaglyptus* is positioned in the superfamily Xanthoidea and the closest species to *E. anaglyptus* is *Leptodius sanguineus.*

Xanthidae is one of the most dominant crab family in the brachyuran with 572 species in 133 genera (De Grave et al. 2009). Although the morphologic identification of xanthids is well studied, mitochondrial genome records of their species are inadequate. There are only one complete mitochondrial genome recorded (Sung et al. 2016). In the present study, we are providing complete mitochondrial genome analysis of a Xanthidae species *Etisus anaglyptus*. Besides, due to the complete mitochondrial genome information, phylogenetic relationship of the species in the superfamily Xanthoidea is investigated. This is the second record for the family Xanthidae and the first record for the genus *Etisus.*

The species have been collected from the rocky intertidal zone of Con Co Island, Vietnam (17° 9′ 54″ N, 107° 20′ 40″ E) on July 2017 and preserved in 97% ethanol. Whole genomic DNA was extracted from walking legs. The methods for sequencing and analyzing complete mitochondrial genome were described previously (Karagozlu et al. 2016). The specimen stored in Department of Biotechnology, Sangmyung University, Korea (SM00247).

Mitochondrial genome length of *E. anaglyptus* is 16,435 bp (GenBank accession number: MG751773). It is composed of 13 protein coding genes, two ribosomal RNA genes, and 22 tRNA genes. The structure and gene orientation of the mitochondrial genome is typical to brachyuran species (Miller et al. 2004). Likewise, all brachyuran records 23 genes encoded on the majority strand and 14 genes positioned in minority strand. The nucleotide distribution of the mitochondrial genome is 33.2% A, 21% C, 11.1% G, and 34.7% T. There are 15 overlapping regions with 1–25 bp in length. The longest overlapping region is located between *nad1* and *tRNA^Leu^*. Besides, the mitochondrial genome has 16 intergenic sequences varying from 1 to 633 bp in length. The largest intergenic sequence is located between 16S rRNA and *tRNA^Val^*. In the mitochondrial genome the genes are using three different initiation codons which are ATA, ATG, and ATT. The most common starting codon is ATG that used by *cytb*, *cox1*, *cox2*, *cox3*, *atp8*, *nad4,* and *nad4l* genes. Also, there are two different stop codons observed which are TAA and TAG.

The phylogenetic study showed that *E. anaglyptus* positioned in the superfamily Xanthoidea and the closest species to *E. anaglyptus* is *Leptodius sanguineus* (KT896744) which is the first complete mitochondrial genome record from the family Xanthidae (Figure 1). Also, the superfamily Xanthoidea has sister group relationship with the superfamily Bythograeoidea. Similar results showed previously by combination of the six nuclear genes and two mitochondrial genes based molecular study (Tsang et al. 2014) and protein coding genes of complete mitochondrial genome based study (Sung et al. 2016). Previously, the genus has been shown to be polyphyletic by three mitochondrial and one nuclear marker based molecular phylogeny study (Lai et al. 2011). Unfortunately, there is no enough mitochondrial genome data recorded to confirm their relationship additional complete mitochondrial genome data from the genus should provide. The present study presents additional data for molecular Xanthidae phylogeny.

**Figure 1.**
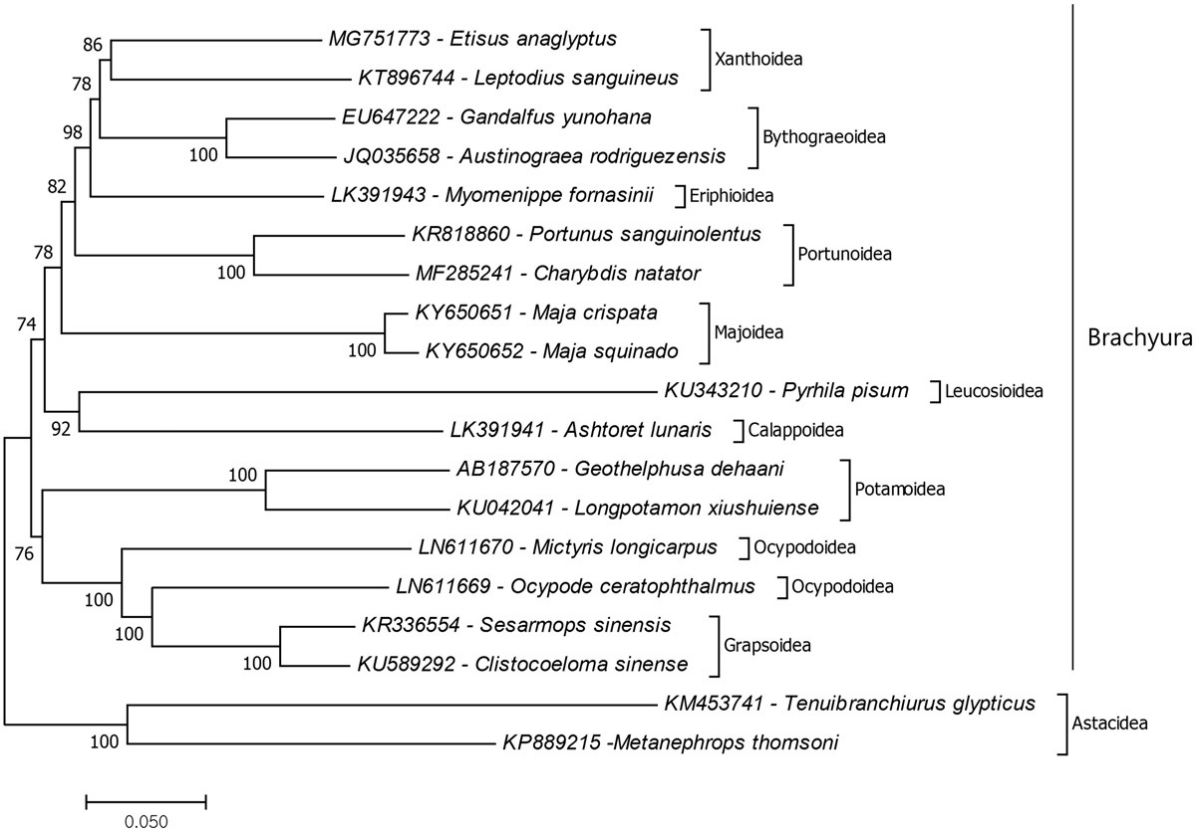
Phylogenetic relationships of that *Etisus anaglyptus* in the infraorder Brachyura due to amino acid sequences of mitochondrial protein coding genes. The mitochondrial genome data which belong to superfamilies of the infraorder Brachyura retrieved from GenBank. Maximum two species chosen as representative of brachyuran super families and the species belongs to the infraorder Astacidea chosen as representative of outgroup. For reconstruction of the phylogenetic tree maximum likelihood statistical method used and bootstrap method replicated 1000 times for the test of phylogeny.
